# Pepsine and Its Incompatibles

**Published:** 1871-04

**Authors:** I. S. Hawley

**Affiliations:** Brooklyn, N. Y.


ARTICLE IV.—*Pepsine and its Incompatibles.* By I. S. HAWLEY, M.D., Brooklyn, N. Y.

Dr. Thomas King Chambers, in his most excellent book on "The Indigestions," speaking of pepsine, says: "But I think that since its introduction to general use, through the ingenious preparation of Dr. Corvisart, it has caused more disappointment than satisfaction. This is because it has been given in unsuitable cases, and because impossible expectations have been founded upon it."

That any one at all acquainted with the physiology of digestion could misapprehend either the indications for the use of pepsine, or found any expectations upon its use, other than the assistance it gives to the digestion of albuminoid articles of food, thereby improving digestion and promoting nutrition, it is difficult to perceive. Yet, doubtless, Dr. Chambers' observation is correct, and a very large proportion of the failures in the administration of pepsine have been due to the causes mentioned by him. Yet there appear to be still other reasons why the use of even true and active medicinal pepsines are unvailing. This is most strikingly illustrated in a short extract from a letter to the Editor of the CHICAGO MEDICAL JOURNAL, which appears in the editorial department of the March number of that journal, under the head of "Chlorate of Potash in Diarrhea and Dysentery."

It is not the object of this communication to notice mixture No. 1, which contains ingredients well known to influence diarrhea and dysentery favorably, without attributing any curative effect to the chlorate of potash; but to call attention to mixture No. 2, which the author denominates "Pepsine Mixture."

Here neither of the errors, mentioned by Dr. Chambers, occur. The case is well chosen, that is, the digestive organs debilitated by diarrhea and dysentery; and the expectation is just, and in accordance with physiological law, "to strengthen and improve the digestive organs and aid digestion." But with a proper object, and a suitable agent for attaining that object, he fails through careless pharmacy, or, perhaps more correctly, disregard of the facts of physiological chemistry, in formulating his prescription. No physiological fact is better established than that alkalies neutralize or destroy the active properties of pepsine. It is well known that the organic principle or ferment called pepsin, is entirely inert when separated from the acid secretion of the stomach, and, for the same reason, becomes inert if the acid be neutralized by the presence of an alkali.

"With the acid there is always present an albuminous matter, which is absolutely necessary to the solvent powers of the gastric juice, and which thence has been named pepsin. It is, in fact, gastric juice without its water and without its acid, and *therefore with its powers dormant, though undestroyed.*"

"It answers our purpose better to view the free acid and the ferment as separate parts of the gastric juice *dependent, indeed, on one another for powers of action*, but not for existence as substances."—Chambers on Digestion and its Derangements.

"The following facts are worthy of notice in reference to the *digestive power* of the gastric juice: it is *suspended* * * * by saturating the free acid with an alkali, or even with the phosphate of lime, by sulphurous, arsenious and tannic acids, * * * and it is *very much impeded by the addition of alkaline salts.*"—Lehman's Physiological Chemistry, vol. i., page 453.

So much for the combination of an alkali with pepsine with the intent to "strengthen and improve the digestive organs and aid digestion."

We next pass to the consideration of the remaining ingredient of the mixture—brandy.

Alcohol is not so much a neutralizer of the active properties of pepsine, as a precipitant of it from its solutions. No chemical law is better known than that chemical action cannot be produced between different substances except one or both be in a state of solution. Hence, if pepsine be precipitated from its solution by alcohol, it cannot act upon the solid contents of the stomach. It is asserted by some that fluids of any alcoholic strength are incompatible with pepsine, though I believe the lighter wines are capable of holding it in solution, and are therefore useful vehicles for its administration. Let us examine the alcoholic strength of this so called "pepsine mixture." Brandy of average quality, sold for medicinal purposes, contains from 48 to 50 per cent. of alcohol. This he dilutes with an equal portion of water, making the mixture contain from 24 to 25 per cent. Now the stronger wines contain only about 10 per cent., and those most suitable for pepsine wine, even less. It therefore appears that had not the pepsine in this mixture been neutralized by an alkali, it would have been precipitated by alcohol and consequently become inert.

It has been thought proper to say thus much upon this subject in the hope to counteract the pernicious effect of a published formula, which is made up of incompatibles. Few things are more taking to a young practitioner than a ready-made formula, which is asserted to have acted successfully. It is sure to be copied and used.

Whatever good this mixture may have done, one thing is sure, the good was not due to its peptic action. Simple pepsine is fully competent to accomplish all the indications here set forth, and if administered simple and alone would doubtless have done so.

Before concluding, it may be well to say a word upon the general subject of administering alkalies in dyspepsia. It is certain they cannot assist digestion, and only serve to arrest an acid fermentation, which is the result of imperfect or prolonged digestion; an imperfection and delay which pepsine would effectually prevent and therefore obviate the necessity for the alkali. Furthermore, it has been clearly proved by recent experiments of M. M. Rabuleau and Constant, that alkalies diminish oxidation and vital force, and induce anemia.

These influences must therefore be doubly damaging where vitality has been lowered by indigestion and consequent innutrition.

